# A Qualitative Study of Factors Influencing Adherence among Pregnant Women Taking Part in a Trial of E-Cigarettes for Smoking Cessation

**DOI:** 10.3390/ijerph18020430

**Published:** 2021-01-07

**Authors:** Allison Ford, Isabelle Uny, Judith Lowes, Felix Naughton, Sue Cooper, Tim Coleman, Peter Hajek, Dunja Przulj, Katie Myers Smith, Linda Bauld, Lesley Sinclair, Robert Walton, Miranda Clark, Michael Ussher

**Affiliations:** 1 Institute for Social Marketing and Health, University of Stirling, Stirling FK9 4LA, UK; isabelle.uny@stir.ac.uk (I.U.); judith.lowes@stir.ac.uk (J.L.); mussher@sgul.ac.uk (M.U.); 2 Behavioural and Implementation Science Group, School of Health Sciences, University of East Anglia, Norwich NR4 7TJ, UK; f.naughton@uea.ac.uk; 3 Division of Primary Care, University of Nottingham, Nottingham NG7 2RD, UK; sue.cooper@nottingham.ac.uk (S.C.); tim.coleman@nottingham.ac.uk (T.C.); Miranda.Clark@nottingham.ac.uk (M.C.); 4 Wolfson Institute of Preventive Medicine, Barts & The London School of Medicine and Dentistry, Queen Mary University of London, London E1 2AT, UK; p.hajek@qmul.ac.uk (P.H.); d.przulj@qmul.ac.uk (D.P.); katie.smith@qmul.ac.uk (K.M.S.); rtwalton123@gmail.com (R.W.); 5 Usher Institute and SPECTRUM Consortium, College of Medicine and Veterinary Medicine, University of Edinburgh, Old Medical School, Teviot Place, Edinburgh EH8 9AG, UK; linda.bauld@ed.ac.uk (L.B.); Lesley.Sinclair@ed.ac.uk (L.S.); 6 Population Health Research Institute, St George’s University of London, London SW17 ORE, UK

**Keywords:** e-cigarettes, vaping, qualitative, interviews, pregnancy, necessity-concerns framework, perceptions and practicalities approach

## Abstract

Use of e-cigarettes (vaping) has potential to help pregnant women stop smoking. This study explored factors influencing adherence among participants in the vaping arm of the first trial of vaping for smoking cessation in pregnancy. We conducted semi-structured telephone interviews (n = 28) with women at three-months postpartum. Interviews were analysed using thematic analysis, informed by the Theoretical-Domains Framework, Necessity-Concerns Framework and Perceptions and Practicalities Approach. Interviewees generally reported high levels of vaping. We found that: (1) intervention adherence was driven by four necessity beliefs—stopping smoking for the baby, and vaping for harm reduction, smoking cessation or as a last resort; (2) necessity beliefs outweighed vaping concerns, such as dependence and safety; (3) adherence was linked to four practicalities themes, acting as barriers and facilitators to vaping—device and e-liquid perceptions, resources and support, whether vaping became habitual, and social and environmental factors; and (4) intentional non-adherence was rare; unintentional non-adherence was due to device failures, forgetting to vape, and personal circumstances and stress. Pregnant smokers provided with e-cigarettes, and with generally high levels of vaping, had positive beliefs about the necessity of vaping for smoking cessation which outweighed concerns about vaping. Non-adherence was mainly due to unintentional factors.

## 1. Introduction

Helping pregnant women to stop smoking is a public health priority. Maternal smoking is a leading preventable cause of morbidity and mortality among pregnant women and infants [1,2,3]. Besides health benefits to herself and her foetus [4], if the woman stops smoking her children are less likely to be exposed to second-hand smoke or to become smokers [5].

There is little evidence for effective interventions for helping pregnant women to stop smoking. Behavioural support provides a modest benefit [6]. Financial incentives show promising results [7]; but are controversial [8]. Bupropion and varenicline are contraindicated in pregnancy [9]. There is evidence for nicotine replacement therapy (NRT) increasing smoking cessation rates in pregnancy, although this effect is not evident when potentially biased, non-placebo-controlled randomsied controlled trials are excluded [4].

Use of electronic cigarettes (EC/vaping) is a potential smoking cessation intervention in pregnancy; there is some evidence for effectiveness in non-pregnant smokers [10,11], and they are increasingly used in pregnancy [12,13], including for cessation [14,15]. In the UK around 5% of pregnant women report vaping [16]. Pregnant women and health professionals (HPs) consider vaping safer than smoking [17,18,19,20], yet also have concerns about safety [14,17,21]. Clinical practice varies; the UK is one of few countries where vaping is advocated as a cessation aid in pregnancy [22]; most other nations advise against vaping in pregnancy (e.g., [23,24]). Evidence outside pregnancy suggests that vaping is likely to be safer than smoking [25]. Safety concerns remain, particularly relating to cytotoxicity and carcinogens [26]. Studies of vaping and health outcomes in pregnancy have equivocal findings [27,28,29,30].

We conducted a qualitative study nested within the first randomised controlled trial of vaping for smoking cessation in pregnancy (PREP trial). Such an intervention is reliant on good levels of adherence. Low adherence is one of the most likely reasons why NRT has failed in pregnancy; factors identified as influencing adherence to NRT include expectations of NRT, experience of using NRT, motivation to stop smoking; beliefs about the necessity of using NRT and safety concerns [31,32]. Qualitative work observes that pregnant women who vape overcome barriers to vaping mainly through having positive beliefs about vaping and becoming proficient at vaping [33]. However, we are not aware of any previous research examining factors influencing adherence among women who have been recommended a vaping intervention. We therefore conducted a theoretically-based, qualitative study with participants in the intervention arm of the PREP trial to explore factors influencing adherence to the vaping intervention. We aimed to: qualitatively explore factors influencing adherence; produce a theoretically-based description of these factors; and consider strategies for maximising adherence.

## 2. Methods

The trial compared usual care (behavioural support plus nicotine patches) with behavioural support plus vaping, among pregnant women willing to receive help to stop smoking (daily smokers, 12–24 weeks gestation, age ≥ 18 years) (https://doi.org/10.1186/ISRCTN62025374). The primary outcome is biochemically validated prolonged abstinence at end of pregnancy. Recruitment ended in November 2019 (N = 1140). Participants in the vaping arm were posted a refillable EC (Innokin Endura T18) and an initial supply of 2 × 10 mL bottles of tobacco flavoured e-liquid (18 mg nicotine) with further supplies provided for up to 8 weeks; where participants disliked the tobacco flavour, fruit flavours were offered. Those finding their e-liquid too strong were offered 11mg e-liquid. Participants received guidance on vaping and support in preparation for the Target Quit Date (TQD), on the TQD, then weekly for four weeks. Participants were encouraged to vape as much as required each day and for as long as they felt they needed to.

To explore vaping adherence, a qualitative descriptive methodology was chosen to elicit an in-depth account of individuals’ views and experiences. We initially determined a sample size target of 30, using the ‘ten plus three’ rule for data saturation. A target of ten participants was set for each of the three vaping/smoking status groups at end of pregnancy (exclusively vaping, dual vaping and smoking, exclusively smoking), to achieve a point where three consecutive interviews have been conducted without new themes emerging. However as most of the women whose details were passed to us from the trial team were dual vaping and smoking we were unable to achieve an even split in this regard. We conducted 28 semi-structured telephone interviews with a convenience sample of participants (February–November 2019). Across the 10-month recruitment period, and following their final trial follow-up at three-months postpartum, consecutive participants in the vaping arm were invited to be interviewed. Fifty-two women agreed to be sent an information sheet and to be contacted. One week later a researcher telephoned the women; texts were sent to alert them to expect the call. Of 43 women we spoke with, 15 declined the interview: four said that they were too busy for an interview; the remaining 11 did not provide a reason. Participants gave verbal consent (recorded digitally) to be interviewed. Interviews were immediately after the consent call or at a more convenient time.

Interviews were arranged and conducted by three female qualitative researchers (AF, IU, JL, non-smokers/vapers) who were separate from the PREP trial team. The interviewers used a topic guide, while allowing participants to speak freely. The topic guide was first informed by the Theoretical Domains Framework (TDF) [34,35] to ensure the broadest coverage of factors influencing adherence. The TDF defines 14 domains into which determinants of health behaviour change, including adherence, can be organised (e.g., social influences, beliefs about consequences, knowledge). The topic guide was then further informed by topics in a study of women’s views of vaping during pregnancy [14], the Necessity-Concerns Framework (NCF) [36] and the Perceptions and Practicalities Approach (PAPA) [37], The NCF proposes that adherence is primarily influenced by necessity beliefs (i.e., judgements of personal need for the treatment) and concerns about the potential adverse consequences of the treatment. The NCF has been used previously to explore pregnant women’s adherence related beliefs about NRT [31]. The PAPA classifies non-adherence as intentional or unintentional and was also a helpful framework for topic guide development around adherence.

Materials were reviewed by two Patient and Public Involvement representatives. Interviews, lasting 20–45 min, were digitally recorded and transcribed verbatim by professional transcribers. Participants received a £20 retail voucher (“Love2Shop”) to compensate them for their time. The study received approval from the Stirling University NHS, Invasive or Clinical Research Ethics Committee (NICR 17/18: 062).

Data were analysed using thematic analysis [38], and focused on the NCF [36] and PAPA [37] to help channel the findings into adherence relevant themes. Analysis was both deductive, informed by these theories and the topic guide, and inductive, from participants’ accounts. Four researchers (AF, IU, MU, JL) reviewed a selection of transcripts and developed a coding framework. Using NVivo12, AF and IU independently coded 10% of randomly selected transcripts from the two smoking status groups (i.e., self-reporting as abstinent or smoking at end of pregnancy). The coding framework was revised and a further 10% of transcripts were independently coded, after which coding consistency was deemed satisfactory, and an analytical framework was established. The remaining transcripts were coded by AF, IU and JL. Using an iterative approach, coded themes were used as the categories for analysis, which were refined, interpreted, labelled and discussed with the wider team until consensus was reached. To indicate the frequency with which themes were provided by participants we used “all”, “almost all”, “most”, “the majority”, “some”, and “a few”. We used the Consolidated Criteria for Reporting Qualitative Research tool [39].

## 3. Results

Interviews were conducted with 20 women self-reporting as smoking and eight self-reporting as abstinent at end of pregnancy (aged 18–48 years) (Table 1 and Table 2). On average, participants had vaped for around seven days a week during the first four weeks of the intervention. At end of pregnancy, EC use remained high: 27 women reported vaping (19 dual using (smoking and vaping) and eight exclusively vaping). Only one woman reported exclusively smoking.

Women’s adherence to vaping was underpinned by both necessity and concern, and practicality issues. We found four necessity beliefs, stopping smoking for the baby, and vaping for harm reduction, smoking cessation and as a last resort, and two main vaping concerns, dependence and safety. These are outlined first below, followed by four practicality themes identified from the data. There was no evidence of divergence of views or experiences between women who were exclusively or dual vaping.

### 3.1. Necessity and Concern Issues

Necessity Beliefs

Women’s reports indicated that vaping adherence was linked to four main necessity beliefs: the need to stop smoking, harm reduction, effective cessation, and vaping as a last resort.

Need to Stop Smoking

Adherence appeared strongly related to the belief that women needed to stop for the baby. This was influenced by awareness, or fear, among all women, that smoking in pregnancy is harmful. This took precedent over any need to stop for their own health. While women described awareness of the harm from smoking to themselves, this was not perceived as different during pregnancy.

“…it wasn’t just about me, to be honest. It was more like trying to do what was best for my baby growing inside of me.”

(Woman13)

Women’s significant others, particularly when they were non-smokers, encouraged this belief by generally being reported to be supportive of women’s cessation attempts through vaping.

Vaping for Harm Reduction

Most women reported the belief that vaping was less harmful than smoking to the baby, “*as far as I was aware [vaping] would be safer for the baby*” (Woman4), and helped them reduce their smoking, “*it helps me to cut down, that’s for sure*” (Woman7). This belief led many women to report vaping beyond end of pregnancy. For many, perceived endorsement of vaping by maternity care HPs, and in particular, introducing the trial to women in a hospital setting, reduced safety concerns and facilitated harm reduction beliefs. For those not having vaped before, who reported little vaping knowledge, this endorsement was even more salient. For a few women this gave them confidence to vape even when others voiced concerns.

“My response [to others] was, and is still now, that like ‘no, the hospital, supported us with this, like, they thought that it’s been a good idea, so that’s why I’m doing it’… because I got it from the hospital… I felt like I was alright to use it.”

(Woman24)

For some, this belief was reinforced through vaping appearing to reduce smoking-related effects.

“I don’t feel anything [vaping]. I don’t feel wheezy, I don’t feel like I need to cough, I don’t feel like my voice is as harsh.”

(Woman14)

For others, this was influenced through less perceived stigma around vaping compared with smoking.

“I think they looked at me a bit better knowing that I was at least trying… When I said I was a smoker they look as if ‘oh god.’ Not a great response, but then when I said ‘I’m actually on the e-cig’, it was like ‘that’s good.’”

(Woman21)

Vaping for Cessation

Almost all women strongly believed that vaping was effective for smoking cessation “*If it weren’t for the e-cigarette, I wouldn’t have stopped smoking”* (Woman28). Some reported this from the early stages of the intervention, often influenced by having observed their own, or significant others’, positive experiences of vaping. The perception among some women that many smokers vaped appeared to normalise vaping.

“A lot of people have done really well on them [to stop smoking] … I kind of knew what they were about and they worked and I had quite high expectations.”

(Woman2)

Many expressed initial doubts about vaping, having had negative experiences.

“I have [vaped before], but obviously I didn’t know how to use them properly. I didn’t know how to change the coil and it didn’t work for me then.”

(Woman15)

However, most of these women reported a growing confidence in vaping as the intervention progressed.

“I’d probably just use the e-cigarette now [for stopping smoking], cos I know I can do it. In the first instance because I wasn’t sure—I wasn’t as confident as I am now.”

(Woman13)

A few continued to believe that vaping could not help them change their smoking.

“I’ve tried it before [for stopping smoking] and it hasn’t worked… For the first few weeks [of the intervention] … I was still smoking cigarettes and I just kind of got to a point where I thought ‘it’s not going to work.’”

(Woman1)

Vaping as a Last Resort

Most women reported that vaping was a last resort to stop smoking. This belief was often influenced by a combination of being motivated to stop for the baby, yet having low confidence in their ability to stop with traditional methods.

“I wanted to stop smoking cos I was pregnant. I just didn’t know how to.”

(Woman15)

Many described quitting as difficult, in and outside of pregnancy, and lacked enthusiasm for pharmacotherapies. Vaping was seen as more appealing than NRT.

“I used patches before and I didn’t like them and I used, you know, nicotine chewing gum before… I didn’t really like that either so the e-cigarette was my last option.”

(Woman25)

“During my other pregnancies I’d been able to give up, but I was really struggling… [vaping] was a lifeline really.”

(Woman21)

### 3.2. Concerns

Women’s concerns around vaping appeared to be less salient than their necessity beliefs. Many reported having no concerns, “*there was very little to worry about, it was more, ‘if I smoke, I’m going to make it worse*’” (Woman28). For some, lack of concern was related to beliefs that EC contained fewer harmful chemicals than cigarettes and that nicotine was not harmful; for others, it related to endorsement by HPs, trial researchers or others.

“I’ve known quite a few people that have smoked an e-cigarette whilst being pregnant as well, and no-one’s sort of every said, ‘oh well, this has happened or that’… there was nothing significant to say, ‘an e-cig can cause this, an e-cig can cause that…’’

(Woman28)

Dependence

The most prevalent concern related to dependence. Some worried that they were vaping too frequently and/or feared long-term addiction. Often influenced by how easily women had taken to vaping, this was a particular concern among those who had not vaped previously and also among those reporting vaping being useful for cessation.

Interviewee: “I thought I was using it too much, do you know, I felt like I was using it constantly through the day, and it was kind of just like, well, I would have only really been out for like one tab [cigarette], but this has been in my hand for an hour.

Researcher: And is that a concern, did that feel worrying to you?

Interviewee: A little bit it did, because I thought, ‘right, I’m over-using it.’” (Woman26)

Many women described their desire to reduce or stop vaping.

“I’ll have to continue to use it for now. Eventually not to have to be this obsessed with it… I would like to not have to use anything.”

(Woman24)

Safety

A few women reported doubts over vaping safety, or the potential for the baby to become addicted to nicotine, although this did not appear to affect adherence, *“I do still question myself, whether, like, it is any good for us or not*” (Woman24). They reported hearing stories about lung issues or exploding devices, from media reports or those around them.

“They keep going on about something called popcorn lung or something like that… something ridiculous… ‘They’re full of chemicals’ and things like that…. It did make me question, but I carried on as long as I could, but it did kind of make me worry.”

(Woman4)

For a few, safety concerns were linked to misperceptions of vaping, that it shouldn’t be used in pregnancy, or limited awareness about different products.

“I didn’t even think I could use an e-cigarette, because I had a bad opinion of an e-cigarette… the ones that you always see with all the train smoke coming out of them? I always think of them as e-cigarettes and I thought ‘oh I don’t know how much smoke the baby can inhale with them.’ I know it’s not inhaling nicotine, but all that smoke in their little lungs… I didn’t realise you could get them slim ones.”

(Woman14)

Some were concerned about the potential inconvenience, “*I didn’t want to upset people by vaping*” (Woman 4), or harm to others, especially children, from vaping indoors or in the car.

“Obviously I wouldn’t [vape] in the car… ‘cause I’ve got my other son in the car… If I was in the car on my own, I would use it… I’m not going to lie… if I’ve come downstairs in the middle of the night to do something for the baby, if he’s upstairs, I’ll have a quick couple while he’s not there.”

(Woman24)

### 3.3. Practicality Issues

Vaping adherence was linked to several practicalities which could act as barriers or facilitators. These included device and accessories, resources and support from the trial and women’s families, habitual vaping, and social and environmental context.

Device and Accessories

The majority of women said they liked the device’s small size, convenience and ease of use (e.g., easy to charge and refill). Some described having it with them always.

“I liked it. It wasn’t too big. You get all different types of e-cigs, but that one is a nice size. It’s one that you can put in our pocket, or you can put it in your bag and it’s comfortable in your hand … It was convenient for me all round.”

(Woman13)

If the device failed, the trial team promptly provided replacements, thereby supporting adherence. For a few women, device failures (e.g., losing charger, flat battery) led to unintentional non-adherence

“I had a problem with the e-cig. I think it was the coil or something. It was leaking anyway. It only took a couple of days for it to come out, so I probably [smoked] about four or five [cigarettes], cos I didn’t have the e-cigarette to smoke.”

(Woman10)

A good supply of e-liquid was crucial. Most women welcomed being sent e-liquid every week, as well as alternatives if they did not like the flavour or strength.

“At first, I didn’t actually like it, I didn’t like the flavour, the liquid that I was given, so that’s why at first I didn’t start using it straight away… they ring you weekly don’t they to see how you’re doing with it, and I explained that I didn’t like it. So, they sent me out new liquids and it was better, so then I started using it pretty much straight away, stopped smoking straight away.”

(Woman24)

A few women said they temporarily continued smoking because they disliked the tobacco flavour sent initially. A few reported disliking all the flavours on offer and purchased their own e-liquid. For most women the nicotine strength provided seemed adequate.

Resources and Support

Most women were satisfied with the advice and support they received from researchers. Hands-on demonstrations were considered particularly useful to those new to vaping. This support promoted adherence because it was considered non-judgmental, non-pressurising and flexible.

“I felt that it was tailored for me because someone used to call me every week and check up on me and I just felt like I was part of something… I thought the ladies who did call me at the time were just very unbiased, non-judgmental.”

(Woman25)

For most, the other main source of support was their partner (with a few partners quitting smoking):

“If it wasn’t for my husband … I would have had a weak moment and I would have probably gone back … I think my husband and my child, unborn child at the time, really just helped me get through...”

(Woman25)

Habitual Vaping

Women seemed most adherent when they managed to make vaping habitual. Vaping offered convenience and ease of use, indoors and outdoors:

“I was just using it when I would have had a normal cigarette though, do you know, part of my routine? So, I had to get up in the morning, make a coffee, go outside, have a cigarette. I was still doing them things but going outside and having the vape.”

(Woman24)

For a few women switching to vaping was difficult, and unintentional non-adherence occurred when the device was not seen:

“It was kind of just remembering that it was there, do you know…I think it was quite easy if it was in plain sight, but if it wasn’t in plain sight or it was on charge, something like that, that’s why it was like, it’s just easier to go for a tab.”

(Woman26)

Social and Environmental Factors

For some women, changes in personal circumstances increased stress and led to unintentional non-adherence:

“I was willing to try but, like I said, because of the stress and the bereavements and obviously having a baby and, sorting my wedding preparations, everything got on top of me and it worked for a while that I was just smoking the e-cig and then, I’m really fussy with food and flavourings and stuff and I just couldn’t find the right flavour. I didn’t stick to it. I was just getting stressed so I started smoking again.”

(Woman17)

A few noted that when they were stressed or busy, especially if they had several children, they forgot to take their EC with them, and smoked.

Being around smokers also led to unintentional non-adherence:

“It was just, you know, when I was at work, and people were smoking, and I wasn’t having a full one of my own, I didn’t have my own, but I would just say, ‘oh, I’ll have some of that’… it wasn’t because I wanted it, it was just because I was outside with everyone that was smoking, with my vape.”

(Woman24)

Only two women described intentional non-adherence, one sometimes preferring cigarettes whilst out and vaping at home; another woman expressed:

“If I went to a family party or somewhere where I know you’re going to get that bit of judgement, I wouldn’t [vape], but the majority of times, say eight times out of ten, I’d use it.”

(Woman13)

## 4. Discussion

This study explored the factors influencing adherence to a vaping intervention in the first trial of vaping for smoking cessation in pregnancy. We found that women’s vaping adherence was influenced by four main necessity beliefs: the need to stop smoking for the baby; vaping for harm reduction; vaping for smoking cessation; and vaping as a last resort. In addition, women’s necessity beliefs outweighed any concerns about vaping, which focused on dependence and safety. Adherence was linked to four practicalities themes relating to the device and e-liquid; resources and support provided; whether vaping became habitual; and social and environmental factors. Examples of intentional non-adherence were rare, while device failures, forgetting to vape, and personal circumstances and stress led to unintentional non-adherence.

The participants in our study, generally, reported high levels of EC usage and positive attitudes to vaping. Other studies have indicated that vaping is appealing to pregnant women who find it challenging to stop smoking via other methods [14,15,40,41,42,43,44]. Unlike other studies, where most women appear to vape only briefly [15,43], our women tended to vape past the intervention stage, until at least end of pregnancy, strongly believing that vaping was helpful. This may be because an extended period of vaping was promoted as part of the intervention. Earlier work has reported uncertainty about the risks of vaping and use of nicotine in pregnancy [20,40,42,45] and women considering vaping ‘worse’ than smoking [40]. For our women, safety concerns were subtler and focused on vaping dependence. Concerns may have been alleviated by information and support provided by the trial and endorsement by hospital staff involved in the study. Yet, there are still concerns about the harmful effects of vaping in pregnancy. Firstly, there are concerns about use of nicotine in ECs. However, ECs share a similar pharmacokinetic profile to fast acting NRT; these concerns are from pre-clinical studies [46,47] and harms have not been found in pregnant NRT users [48,49]. The main concerns are for the vaping of non-nicotine ingredients, such as propylene glycol, glycerine, flavourings and other additives. Several studies have examined the effects of vaping on health outcomes in pregnancy; these studies have equivocal findings and they all have major methodological limitations, such as not assessing the extent of use of ECs [27,28,29,30]. More rigorous studies are needed, including research which quantifies the level of toxicants and carcinogens associated with vaping in pregnancy, as has been done with non-pregnant individuals [50]; findings may be different for pregnant versus non-pregnant populations, for example, due to higher metabolism in pregnancy [51] leading to possible increased levels of vaping. Moreover, the long-term of health effects of vaping compared with smoking, including respiratory effects [52], are not known.

Unlike some previous studies, there were few reports of perceived stigma around vaping [14,33]. Safety concerns and perceived stigma may have been more evident if we had interviewed those with lower EC usage.

The findings indicate several strategies for maximising adherence to vaping interventions in countries that support this route, such as the UK, and for women who have not been able to quit using traditional approaches. These strategies have implications for practice, i.e., they may be relevant to health professionals in clinical interactions with women who smoke in pregnancy, and for the development of cessation interventions for pregnant women. First, women need increased awareness that vaping is a viable harm reduction and/or cessation tool. Given the importance of HPs in women’s antenatal care and smoking cessation [44], assisting HPs to communicate benefits of vaping in pregnancy relative to smoking may give women greater confidence in vaping. Peer assistance may also play a role, given the value women placed on peer accounts of vaping. Second, adherence may be maximised through addressing women’s concerns, particularly around overuse or long-term vaping; it may be useful for women to know of any support available for stopping vaping. Women’s concerns around second-hand vapour, particularly on children, have been noted elsewhere [14]. Interventions should adequately address misleading media narratives and provide guidance on vaping around others. Third, it is important that vaping interventions, or advice on vaping, are delivered by practitioners knowledgeable about products, considering convenience, ease of use, durability and women’s preferences [14]. As with traditional, effective cessation interventions, which are characterised by extended support and follow-up [53], our findings suggest vaping interventions should provide continuing, regular technical and behavioural support, delivered in a non-judgemental but informed manner, and responsive to social and contextual factors. It is unclear how effective vaping is as an intervention if delivered without behavioural support [54]. Regular communication with women, for example through the use of apps or text messaging, may help to address some of the factors leading to unintentional non-adherence (e.g., forgetting to vape), or may alert those delivering interventions to device issues which can be quickly resolved.

Well-designed vaping interventions, which maximise adherence, would likely benefit women over impulsive EC purchases commonly reported, where decisions are based on cost or family/friend recommendations and women feel uninformed about vaping [14]. Research should explore which interventions are most effective for maximising adherence, for example, whether digital support for pregnant women can increase adherence, and whether women’s lifestyle or sociodemographic factors contribute to adherence levels. The latter issue will be examined as part of the results for the main trial in which this qualitative study is nested. There is also a need to investigate whether increased vaping adherence translates into improved smoking outcomes and this will also be reported elsewhere in our main findings for the trial.

A strength of the study is the use of a theoretically-based topic guide and analysis approach, informed by theories relevant to adherence. Together, we found the NCF and PAPA frameworks useful, complimentary, and extensive for adherence analysis. Structuring adherence around women’s beliefs and concerns via the NCF proved particularly insightful for understanding women’s influences. We found it challenging to incorporate social, environmental and interpersonal factors within these frameworks, which may need to be extended to accommodate these factors, or a social-ecological framework [55] may need to be added.

The study has limitations. Overall, our sample had high levels of EC use and positive attitudes to vaping. This is unsurprising given women were recruited through a vaping trial and those with positive experiences and good levels of vaping adherence may have been more likely to agree to be interviewed. A key limitation is that we were unable to learn about adherence from women who reported low EC use. While we have learnt about some of the factors leading to high adherence, we therefore have an absence of data identifying factors of low adherence. It is possible that a study which includes a greater number of women with low EC use may identify other factors related to adherence, including greater concern about vaping. Further studies are therefore needed among pregnant women who have been advised to vape and report poor adherence or discontinue treatment prematurely. That our results found a dominance of necessity beliefs over concerns may be, again, due to an inherent sample bias, as nearly all were still vaping at end of pregnancy and held favourable opinions about vaping. It is possible that UK policies on harm reduction and the UK research context contributed to favourable opinions towards vaping [25]. Our findings are specific to adherence within a trial context, where women received ongoing support and regular follow-ups. However, as similar support would be provided if vaping was rolled out within the UK National Health Service as a cessation treatment during pregnancy, there is no reason why adherence, or the factors which influence it, may be substantially different to our study in clinical practice. Although representativeness was not the intention of this qualitative study, the findings may not apply to wider populations of women in the UK or elsewhere. Finally, our sample also lacks ethnic diversity, although in this regard it is fairly typical of women who smoke in pregnancy in the UK (i.e., mostly white ethnicity) [56].

## 5. Conclusions

To our knowledge this is the first study to examine the factors influencing vaping adherence within a trial. Pregnant women, with generally high levels of EC use, had strong beliefs about the necessity of vaping, which outweighed any concerns about vaping. Non-adherence was mainly due to unintentional factors, such as device failures, forgetting to vape and stress, which need to be addressed to prevent relapse to smoking. Overall, women had positive attitudes towards vaping and reported few barriers to the use of e-cigarettes for smoking cessation.

## Figures and Tables

**Table 1:** Participant characteristics at trial primary endpoint at end of pregnancy (n = 28).

	No. (%)
Age (years)	
Mean (SD) (range)	28.6 (6.65) (18–48)
Ethnicity	
White British	25 (89.3)
White European	1 (3.6)
Asian British	1 (3.6)
Black British	1 (3.6)
Self-reported smoking and vaping status at end of pregnancy	
Exclusively smoking	1 (3.6)
Smoking and vaping	19 (67.9)
Exclusively vaping	8 (28.6)
EC use in trial intervention phase *	
Mean (SD) days	6.6 (1.44)

* EC use = mean (SD) number of days a week that women vaped over the first four weeks after the quit date. Weekly data was missing for three participants. For four participants, data was available for three weeks and has been calculated over this time frame.

**Table 2:** Individual participant characteristics.

Participant ID	Age	Ethnicity	Smoking Status at End of Pregnancy	Vaping Status at End of Pregnancy	EC Use * Mean (SD) Days
Woman1	23	White British	Smoking	Vaping	7.0 (0)
Woman2	25	White British	Smoking	Vaping	Not known
Woman3	31	White British	Abstinent	Vaping	7.0 (0)
Woman4	29	White British	Smoking	Not vaping	7.0 (0)
Woman5	37	White British	Smoking	Vaping	5.8 (2.50)
Woman6	24	White British	Abstinent	Vaping	5.3 (2.06)
Woman7	31	White European	Smoking	Vaping	7.0 (0)
Woman8	18	White British	Smoking	Vaping	7.0 (0)
Woman9	33	White British	Smoking	Vaping	5.8 (2.5)
Woman10	32	White British	Smoking	Vaping	7.0 (0)
Woman11	27	White British	Smoking	Vaping	7.0 (0)
Woman12	25	White British	Smoking	Vaping	6.5 (1.00)
Woman13	39	Black British	Abstinent	Vaping	7.0 (0)
Woman14	29	White British	Smoking	Vaping	Not known
Woman15	27	White British	Smoking	Vaping	7.0 (0)
Woman16	48	White British	Abstinent	Vaping	7.0 (0)
Woman17	29	White British	Smoking	Vaping	3.0 (2.94)
Woman18	20	White British	Smoking	Vaping	7.0 (0)
Women19	20	White British	Smoking	Vaping	7.0 (0)
Woman20	25	White British	Smoking	Vaping	7.0 (0)
Woman21	40	White British	Abstinent	Vaping	7.0 (0)
Women22	26	White British	Abstinent	Vaping	7.0 (0)
Woman23	29	White British	Smoking	Vaping	Not known
Woman24	30	White British	Abstinent	Vaping	7.0 (0)
Woman25	32	Asian British	Smoking	Vaping	7.0 (0)
Woman26	21	White British	Smoking	Vaping	6.7 (0.58)
Woman27	23	White British	Abstinent	Vaping	7.0 (0)
Woman28	28	White British	Smoking	Vaping	7.0 (0)

* EC use = mean (SD) number of days a week that women vaped over the first four weeks after the quit date. For women 7, 10, 25 and 26, data was available for three weeks and has been calculated over this time frame.

## Data Availability

Data sharing is not applicable to this article.
